# Mapping technological innovation dynamics in artificial intelligence domains: Evidence from a global patent analysis

**DOI:** 10.1371/journal.pone.0262050

**Published:** 2021-12-31

**Authors:** Na Liu, Philip Shapira, Xiaoxu Yue, Jiancheng Guan

**Affiliations:** 1 School of Management, Shandong Technology and Business University, Yantai, China; 2 Manchester Institute of Innovation Research, Alliance Manchester Business School, University of Manchester, Manchester United Kingdom; 3 School of Public Policy, Georgia Institute of Technology, Atlanta, Georgia, United States of America; 4 School of Public Policy and Management, Tsinghua University, Beijing, China; 5 School of Economics and Management, University of Chinese Academy of Sciences, Beijing, China; The Open University of Hong Kong, HONG KONG

## Abstract

Artificial intelligence (AI) is emerging as a technology at the center of many political, economic, and societal debates. This paper formulates a new AI patent search strategy and applies this to provide a landscape analysis of AI innovation dynamics and technology evolution. The paper uses patent analyses, network analyses, and source path link count algorithms to examine AI spatial and temporal trends, cooperation features, cross-organization knowledge flow and technological routes. Results indicate a growing yet concentrated, non-collaborative and multi-path development and protection profile for AI patenting, with cross-organization knowledge flows based mainly on interorganizational knowledge citation links.

## Introduction

Artificial intelligence (AI) involves the creation of machines or agents that seek to simulate human rationality [1–3]. AI may use machine learning, neural networks, deep learning, natural language processing, and other information technologies to imitate or augment human capabilities, through logical calculation or through cognitively modelling human consciousness [4, 5].

AI is a rapidly growing and cross-disciplinary domain [4, 6]. There is an expectation that AI will be a key driver of future economic development [7]. The global AI market size, valued at US $ 27.2 billion in 2019, is projected to reach US $ 266.9 billion by 2027 [8]. Bundled with other information and automation technologies, AI is a key enabler of what is seen as a rapidly evolving digital transformation phenomenon that is now disrupting and challenging multiple aspects of business and society and driving organizational transformation and strategic change [9–11]. As a general-purpose technology, AI is applied increasingly in areas as diverse as power electronics, transportation, healthcare, manufacturing, finance, and education [2, 5, 9, 12]. Governments are actively advancing AI technologies for economic, societal, environmental, and security purposes, with the OECD [13] identifying more than 600 policy initiatives in 60 countries and jurisdictions. Measures adopted include national AI strategies, increased R&D and training, the formation of public-private intermediary organizations, and (with variations by country) new governance and regulatory guidelines. Accompanying these private and governmental efforts are concerns about AI’s ethical, personal privacy, employment, societal, distributional, and political dimensions [14, 15].

The growth of AI—and the multiple consequences and concerns associated with its development—make it important to probe the innovation dynamics and evolution of the domain. Among the various ways of understanding AI trends, one approach is to investigate patent activity to ascertain efforts (at national and organizational scales) to exploit AI intellectual property rights and fields and applications of interest. While patents are primarily intended to protect inventions in return for disclosure, the use of patents as a proxy for innovation activity is a well-established practice in innovation management and policy studies [16]. Not all inventions are patented and not all granted patents are of equal economic value or necessarily lead to successful innovations. Nonetheless, indications of technological development and insights about the direction and pace of innovation efforts can be obtained through the quantitative analysis of patent applications including through the profiling of inventors, owners, organizational types, locations, technological content, citations, collaborations, and other information contained in records of patenting activity [17–21].

Among examples in the extant literature of studies that use patent data to probe AI technology and innovation developments, Tseng and Ting [22] used patent quantity and quality measures to explore AI technology trends. Just over 5,200 US AI patent grants were identified from 1976 through to 2010, using a single technological class (Data Processing: AI) sub-divided into four AI fields (problem reasoning and solving, machine learning, network structure, and knowledge processing). China was not discussed as, in this early study, it did not rank among the top ten leading countries for AI patenting (in the US data). Fujii and Managi [23] examined AI technology shifts using a patent decomposition framework. This study covered global developments from 2000 through to 2016, identifying about 13,500 AI patent grants. There is again a narrowly targeted search approach on a single patent group (computer systems based on specific computation models), with the finding of a shift in AI patenting from biological and knowledge-based approaches to mathematical and other models. Van Roy et al. [4] used text-mining to map the global AI patent landscape, highlighting emerging AI technologies and hotspots across the world. Using a keyword approach (49 AI-related terms), this study identified 155,000 patent family applications worldwide from 2000 through to 2016, of which 36.4% (about 56,000) had been granted. The search strategy captured AI-related patent records beyond those classified in computing or data processing patent groups. The growth of AI patenting in China through to 2016 is described. However, the keyword search strategy is not thoroughly explained.

In recent years, as we will see, the domain of AI—and AI patenting—has seen a further acceleration in scale and scope, with new technological and functional approaches emerging. Hence, while the existing work provides useful insights, the rapidly evolving nature of AI creates both a need and an opportunity for search approach refinement and further updated analysis. In this paper, we advance a new search strategy for identifying AI-oriented patent documents that captures this rapidly multiplying domain with high recall and precision. After explaining its construction, we apply this search strategy to build a comprehensive picture at country, organization, and technology levels. We integrate patent analytics with network and main path analysis to address questions about where AI technological knowledge is located, who are the leading organizations developing AI over successive time periods, and what are the most active technologies and technological development routes in the AI domain. Our aim is to offer a public AI patent document search approach and to provide insights for policy and management.

## Materials and methods

### Data collection

A key task in quantitatively profiling the development of patenting in an emerging technology is to identify, with high recall and precision, which patent documents relate to the focal field [24, 25]. However, there is no agreed definition as to the composition of AI patenting. Indeed, the emerging AI domain, which intrinsically involves novelty, boundary-crossing, and fast growth, generates challenges in identifying patents in this field [4, 6].

Some researchers have adopted AI patent search strategies based on designated patent classification codes including the International Patent Classification (IPC) [22, 23]. While easily implemented, this approach overlooks AI-related patents in classes outside of the designated codes and, as patent classifications tend to change slowly, does not match AI’s rapid technological evolution especially in recent years. To address such challenges, other researchers have used a keyword-based search [4], or a combination of these two search approaches [5, 26]. A combined approach has the advantage of improving recall for patents in patent classes that are predominantly AI-related, but which might not otherwise be captured by specific keywords. However, to be most effective, there needs to be systematic selection and testing of designated keywords and patent classifications.

In this study, we combine a systematic keyword-based search with IPC and CPC (Cooperative Patent Classification) codes to select AI-related patents. (See Table 1.) The keywords are from our previous peer-reviewed bibliometric definition of AI constructed from benchmark AI publications; this approach has relatively high recall and precision in capturing AI-related publications [6]. We then manually selected and tested AI-specific CPC and IPC codes (Table 1). We identify a patent document as AI-related if its title, abstract or claims matched at least one AI keyword, or it was assigned at least one of the CPC or IPC AI codes. Keyword searching in the patent title, abstract and claims has been found to be an effective strategy for ensuring optimal recall and precision [19] and is consistent with other patent studies of technological trends [20]. (For further details, including text descriptions of the selected patent codes, see S1 Appendix).

**Table 1:** Search strategy for artificial intelligence patents.

No.	Search terms
# 1 keyword search	Title Abstract Claims = (artificial intelligen* OR neural net* OR machine* learning OR expert system% OR natural language processing OR deep learning OR reinforcement learning OR reinforced learning OR learning algorithm% OR *supervised learning OR intelligent agent* OR back_propagation learning OR Bp learning OR back_propagation algorithm* OR long short-term memory OR (Pcnn% AND NOT Pcnnt) OR pulse coupled neural net* OR Perceptron% OR neuro_evolution OR liquid state machine* OR deep belief net* OR radial basis function net* OR Rbfnn* OR Rbf net* OR deep net* OR autoencoder* OR committee machine* OR training algorithm% OR back_propagation net* OR bp network* OR Q learning OR convolution* net* OR actor-critic algorithm% OR feed_forward Net* OR hopfield net* OR neocognitron* OR xgboost* OR boltzmann machine* OR activation function% OR neuro_dynamic programming OR learning model* OR neuro_computing OR temporal difference learning OR echo state* net* OR transfer learning OR gradient boosting OR adversarial learning OR feature learning OR generative adversarial net* OR representation learning OR multi_agent learning OR reservoir computing OR co-training OR Pac learning OR probabl* approximate* correct learning OR extreme learning machine* OR ensemble learning OR machine* intelligen* OR neuro_fuzzy OR lazy learning OR multi* instance learning OR multi_instance learning OR multi* task learning OR multi_task learning OR computation* intelligen* OR neural model* OR multi* label learning OR multi_label learning OR similarity learning OR statistical relation* learning OR support* vector* regression OR manifold regulari?ation OR decision forest* OR generali?ation error* OR transductive learning OR neuro_robotic* OR inductive logic programming OR natural language understanding OR adaboost* OR adaptive boosting OR incremental learning OR random forest* OR metric learning OR neural gas OR grammatical inference OR support* vector* machine* OR multi* label classification OR multi_label classification OR conditional random field* OR multi* class classification OR multi_class classification OR mixture of expert* OR concept* drift OR genetic programming OR string kernel* OR learning to rank* OR machine-learned ranking OR boosting algorithm% OR robot* learning OR relevance vector* machine* OR connectionis* OR multi* kernel% learning OR multi_kernel% learning OR graph learning OR naive bayes* classifi* OR rule-based system% OR classification algorithm* OR graph* kernel* OR rule* induction OR manifold learning OR label propagation OR hypergraph* learning OR one class classifi* OR intelligent algorithm*)
# 2 CPC search	CPC = (A61B 5/7264, A61B 5/7267, A63F 13/67, B23K 31/006, B25J 9/161, B25J 9/163, B29C 66/965, B29C2945/76946, B29C2945/76949, B29C2945/76979, B60G2600/1876, B60G2600/1878, B60L2260/46, B60T 8/174, B60T2210/122, B64G2001/247, B65H2557/38, B66B 7/043, B66B 7/045, E21B2041/0028, F01N2900/0402, F02D 41/1405, F03D 7/046, F05B2270/709, F05D2270/709, F16H2059/086, F16H2061/0084, F16H2061/0087, G01N 29/4481, G01N 30/8662, G01N 33/0034, G01N2201/1296, G01R 31/2846, G01R 31/3651, G01S 7/417, G05B 13/027, G05B 13/028, G05B 13/0285, G05B 13/029, G05B 13/0295, G05B 23/0229, G05B 23/024, G05B 23/0254, G05B 23/0281, G05B2219/13111, G05B2219/13166, G05B2219/21002, G05B2219/23253, G05B2219/23288, G05B2219/24086, G05B2219/25255, G05B2219/31351, G05B2219/31352, G05B2219/31353, G05B2219/31354, G05B2219/32193, G05B2219/32327, G05B2219/32329, G05B2219/32334, G05B2219/32335, G05B2219/33002, G05B2219/33013, G05B2219/33014, G05B2219/33015, G05B2219/33021, G05B2219/33024, G05B2219/33025, G05B2219/33026, G05B2219/33027, G05B2219/33028, G05B2219/33029, G05B2219/33033, G05B2219/33034, G05B2219/33035, G05B2219/33038, G05B2219/33039, G05B2219/33041, G05B2219/33044, G05B2219/33056, G05B2219/33065, G05B2219/33066, G05B2219/33295, G05B2219/33303, G05B2219/33321, G05B2219/33322, G05B2219/34066, G05B2219/34081, G05B2219/34082, G05B2219/36039, G05B2219/36456, G05B2219/39071, G05B2219/39072, G05B2219/39095, G05B2219/39268, G05B2219/39271, G05B2219/39276, G05B2219/39282, G05B2219/39283, G05B2219/39284, G05B2219/39286, G05B2219/39292, G05B2219/39294, G05B2219/39297, G05B2219/39298, G05B2219/39311, G05B2219/39312, G05B2219/39352, G05B2219/39372, G05B2219/39374, G05B2219/39376, G05B2219/39385, G05B2219/40107, G05B2219/40115, G05B2219/40408, G05B2219/40494, G05B2219/40496, G05B2219/40499, G05B2219/40528, G05B2219/40529, G05B2219/41054, G05B2219/42018, G05B2219/42135, G05B2219/42141, G05B2219/42142, G05B2219/42149, G05B2219/42287, G05B2219/49065, G05D 1/0088, G05D 1/0221, G06F 7/023, G06F 11/1476, G06F 11/2257, G06F 11/2263, G06F 15/18, G06F 16/243, G06F 16/24522, G06F 16/3329, G06F 16/3344, G06F 16/90332, G06F 17/20, G06F 17/2282, G06F 17/28, G06F 17/2881, G06F 17/289, G06F 17/30401, G06F 17/3043, G06F 17/30654, G06F 17/30684, G06F 17/30976, G06F 19/24, G06F 19/345, G06F 19/707, G06F2207/4824, G06K 7/1482, G06K 9/6256, G06K 9/6264, G06K 9/6269, G06K 9/627, G06K 9/6273, G06N 3/004, G06N 3/008, G06N 3/02, G06N 3/0427, G06N 3/0445, G06N 3/0463, G06N 3/0481, G06N 3/049, G06N 3/06, G06N 3/08, G06N 3/084, G06N 3/086, G06N 5, G06N 5/00, G06N 5/02, G06N 5/043, G06N 7/023, G06N 7/046, G06N 20, G06N 20/00, G06N 20/10, G06N 20/20, G06N 99/005, G06T 3/4046, G06T 9/002, G06T2207/20081, G06T2207/20084, G07C2009/00849, G07C2009/00888, G07D 7/2083, G08B 29/186, G08G 1/096888, G10H2250/311, G10K2210/3024, G10K2210/3038, G10L 15/06, G10L 15/144, G10L 15/16, G10L 15/18, G10L 17/18, G10L 25/30, G11B 20/10518, G16B 40, G16C 20/70, G16H 50/20, G21D 3/007, G21D2003/007, H01H2009/566, H01H2047/009, H01J2237/30427, H01M 8/04992, H02H 1/0092, H02P 21/0014, H02P 21/0025, H02P 23/0018, H02P 23/0031, H03H2017/0208, H03H2222/04, H04L 12/2423, H04L 25/0254, H04L 25/03165, H04L 41/16, H04L 45/08, H04L 45/36, H04L2012/5686, H04L2025/03464, H04L2025/03554, H04N 21/4662, H04N 21/4663, H04N 21/4665, H04N 21/4666, H04Q2213/054, H04Q2213/13054, H04Q2213/13343, H04Q2213/343, H04R 25/507, Y10S 128/924, Y10S 128/925, Y10S 706)
# 3 IPC search	IPC = (A63F 13/67, G06F 8/33, G06F 15/18, G06F 17/20, G06F 17/21, G06F 17/27, G06F 17/28, G06F 19/24, G06K 9/66, G06N 3/02, G06N 3/04, G06N 3/06, G06N 3/063, G06N 3/067, G06N 3/08, G06N 3/10, G06N 5/00, G06N 5/02, G06N 5/04, G06N 7/02, G06N 20, G06N 20/00, G06N 20/10, G06N 20/20, G06T 1/40, G10L 15/06, G10L 15/16, G10L 15/18, G10L 17/04, G10L 17/10, G10L 17/18, G10L 25/30, G16B 40, G16B 40/00, G16B 40/20, G16B 40/30, G16C 20/70, G16H 50/20, H01M 8/04992)
# 4 AI patents	# 4 = # 1 OR # 2 OR # 3

We applied our AI patent search strategy to PatentSight—a comprehensive worldwide patent database covering more than 100 million patent documents from over 95 authorities including the European Patent Office, the US Patent and Trademark Office, and national patent offices around the world [27]. Our search (based on the approach in Table 1) identified 383,168 AI patent families (all years through to May 7, 2020) comprising applications and grants (excluding design and plant patents and utility models from China).

### Methodological approach

We track AI patent documents by their application years. Since patent granting takes time (up to two years for US patents), the application year is closer to the period when the patent was developed [28]. To avoid double counting, we analyze patent families rather than individual patent documents. A patent family is a collection of patents filed in several patent offices to protect the same invention [4, 29]. While we summarily report on patent grants, noting where there are major differences compared with applications, our analysis uses patent applications which are useful for detailed consideration of topical technological trends (as opposed to assessments of patent economic value) [30]. Patents applied for reflect R&D efforts, new technological opportunities, innovative capacities, and future performance potentials [31, 32]. We use patents applications in the analysis of country and organizational performance and technological trends and development routes. Drawing on Frietsch and Schmoch [33], we use counts of transnational patent applications filed at the European Patent Office (EPO) or international applications filed under the Patent Cooperation Treaty (PCT) to capture high-quality patents. To assign patents to countries, we use inventor location. We fractionally count patent documents with multiple inventors from different countries [34]. As a proxy for multi-country inventor collaboration, we compute international co-patent documents in a certain country as those with at least one inventor located abroad. Based on international co-patenting, we map collaborative network relationships among leading countries [35].

We homogenized assignee names using VantagePoint software to address syntax variants for the same patent assignee and to identify transnational patent documents. To delineate interorganizational knowledge flows, we identified co-patenting relationships based on patent documents with multiple assignees [36, 37] and analyzed forward citation networks [38]. Forward citations—the citations of an organization’s patents by other organizations—are often used to evaluate organizational knowledge diffusion [38, 39]. In interorganizational citation networks, nodes refer to organizations and edges refer to the directed links obtained through citing and the cited relationship between organizations.

We further developed citation-based indicators to measure the openness of an organization’s knowledge flow in the processes of its patented innovation, which can be divided into the dimensions of outward diffusion, inward absorption, and self-creation. An organization’s knowledge diffusion capacity, reflecting its knowledge spillover or knowledge contribution to other organizations, is computed through the formula:

$$TDC_{i}=\frac{\overset{\text{n}}{\underset{j=1}{\sum }}k_{ij}-k_{ii}}{\overset{m}{\underset{i=1}{\sum }}\overset{n}{\underset{j=1}{\sum }}k_{ij}}$$ (1)

where *k*_*ij*_ is the number of weighted directed citation links from node *i* to node *j*; *k*_*ii*_ has a similar meaning, the numerator denotes knowledge outflow of node *i*, and the denominator is the total knowledge outflow of all nodes. In the same way, knowledge absorptive capacity representing knowledge gains of an organization from other organizations can be defined as:

$$TAC_{i}=\frac{\overset{\text{n}}{\underset{j=1}{\sum }}k_{ji}-k_{ii}}{\overset{m}{\underset{i=1}{\sum }}\overset{n}{\underset{j=1}{\sum }}k_{ji}}$$ (2)

where the numerator denotes knowledge inflow to node *i* and the denominator is the total knowledge inflow to all nodes. Technology absolute impact can be calculated by formula: *TAI*_*i*_ = *TDC*_*i*_ + *TAC*_*i*_, which is a measure of overall knowledge capacity of node *i*, including knowledge diffusion and knowledge absorption. The greater the technology absolute impact of an organization, the more knowledge flows through it, indicating that the organization acts as a knowledge broker.

Finally, we performed analyses at the technology level looking at the identity of technological building blocks and their development paths in the AI domain. We focused on the most frequently occurring AI IPC classes at 4-digit levels and finer granularities. Each patent document is assigned one or more IPC classes, allowing analysis of detailed AI technologies as captured by the IPC taxonomy system. We identified the patent level citation network based on forward citations in the dataset to identify relationships from focal to other patents [40]. In this patent citation network, nodes refer to patents and edges refer to directed links obtained through the citing and cited relationship. We then applied a main path analysis method to the patent citation network to identify AI technological development trajectories. Main path analysis was introduced by Hummon and Dereian [41] and extended by Verspagen [42] and Liu and Lu [43]. Main path analysis first calculates the traversal weight for every link in the citation network and then seeks the main path based on the traversal weights using a specific search algorithm [44]. Traversal weight is the times that a link is traversed through searching from starting nodes to ending nodes of a citation network [45]. There are several algorithms available to calculate traversal weight, involving search path count (SPC), search path link count (SPLC), search path node pair (SPNP) and node pair projection count (NPPC) [41, 42]. In this study, we applied SPLC to calculate traversal weight as it reflects knowledge diffusion scenarios in science and technology development [44]. Algorithms available for identifying main technological trajectories include local main path, global main path, and key-route main path [43], each of which tends to produce similar results [46]. We applied the key-route (global) search algorithm using Pajek software for our investigation of AI technological trajectories.

## Results

**Fig 1:**
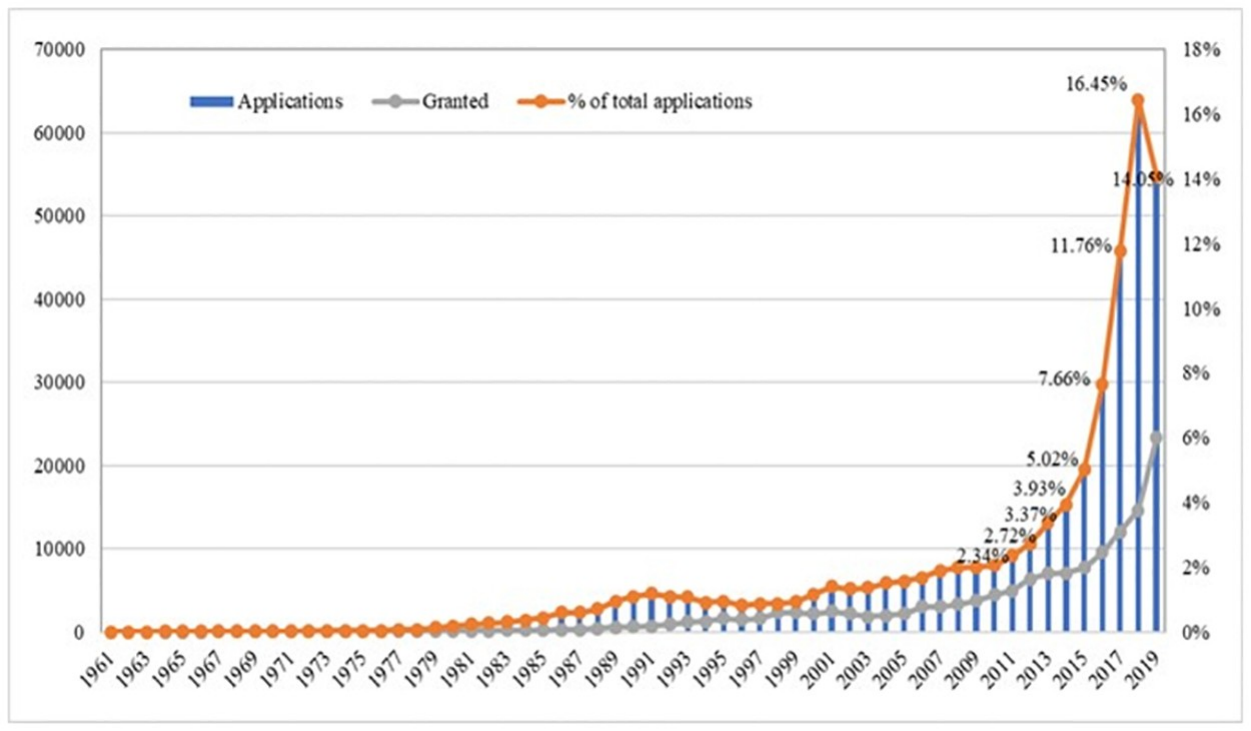
AI patent applications and grants by year.

AI patent applications started to grow in the early 1980s, with modest yet fluctuating growth through to the early 2010s (Fig 1). In the 2010s, AI patent applications entered a rapid expansion. From 2011 to 2018, AI patent applications averaged 30 percent annual growth, with a remarkable average annual growth of 48 percent between 2015 and 2018. Over half of total AI patent applications were filed in the most recent five years. The apparent drop-off of patent applications in 2019 is due to the time gap between patent filing and publishing. Patent grants broadly follow these trends, albeit at a lower level.

### Country-level landscape

#### Trends by AI patent productivity and quality

Assigned by inventor location (with fractional counts for multi-inventor patents), ten leading countries together contributed 95% of worldwide AI patent applications and 93% of grants. Through to 2019, patent grants comprised about 39% of all patent applications worldwide. China now has the greatest share of patent applications (41.2%), followed by Japan (20.5%), the USA (20.3%), South Korea (5.1%), Germany (2.3%) and the UK (1.3%). (Table 2.) Currently, Chinese pending applications account for nearly 73% of total pending AI patent applications worldwide, reflecting China’s considerable efforts to develop AI in recent years [47, 48]. However, China has a less significant presence in the granted and transnational patent landscape (21.3% and 10.1% respectively). The USA ranks first both in terms of granted patents (32.6%) and transnational patents (39.2%), highlighting its established leadership and level of patent quality. Japan has a 21.3% share of granted patents and a 13.1% share of transnational patents. Other countries with positions in the granted and transnational AI patent landscape are South Korea, Germany, and the UK, with shares of 7.6%, 3.0% and 1.8% in granted patents and 4.3%, 6.8% and 3.9% in transnational patents, respectively. International collaboration among inventors is particularly high for India, the UK, and Canada.

**Table 2:** AI patents by countries.

	Patent applications									Patent grants			
Country	All		Pending		Transnational		International co-pat.			All		Cited top 10%	
	N	%	N	%	N	%	N	%	% of apps	N	%	% All	% 2015–2019*
China	158.0	41.2	109.9	72.6	7.7	10.1	3.2	15.7	2.0	31.5	21.3	2.1	19.2
USA	77.8	20.3	16.8	11.1	29.9	39.2	14.9	73.9	19.1	48.1	32.6	69.5	56.5
Japan	78.6	20.5	8.0	5.3	10.0	13.1	1.2	5.9	1.5	30.0	20.3	9.9	2.6
South Korea	19.6	5.1	3.8	2.5	3.3	4.3	1.0	5.1	5.2	11.7	7.9	1.2	2.2
Germany	9.0	2.3	2.5	1.7	5.2	6.8	2.7	13.3	29.9	4.4	3.0	2.1	2.1
UK	4.8	1.6	1.3	0.8	3.0	3.9	2.7	13.5	56.4	2.6	1.8	2.7	2.7
India	4.5	1.2	1.5	1.0	1.6	2.1	2.8	13.9	61.9	2.2	1.5	1.0	1.9
Canada	4.5	1.2	1.1	0.7	2.0	2.7	2.4	11.7	52.5	2.4	1.6	2.7	3.0
Taiwan	4.0	1.0	0.5	0.3	0.2	0.3	0.9	4.4	21.9	2.6	1.8	0.3	0.5
France	3.6	1.0	0.7	0.5	2.3	3.1	1.4	6.8	37.5	2.4	1.6	1.4	1.2
Total	383.2	100.0	151.4	100.0	76.3	100.0	20.1	100.0	5.3	147.8	100.0	100.0	100.0

Source: Analysis of PatentSight patent documents as of May 7, 2020, using patent search approach (see text). Numbers (N) in thousands.

Note:

*Granted in years 2015–2019.

By citations received to AI granted patents, which can be considered (with qualifications) as a proxy measure of patent value and technological importance [16], the USA has a dominant position. US inventors accounted for about 30% of granted AI patents but almost 70% of the most cited (top 10% cited) patents. (Table 2.) Canada and the UK also have a relative higher presence in the top 10% of cited AI patents relative to their shares of granted AI patents. China contributes just over 2% of the top 10% of cited patents overall (compared to more than 20% of all AI patent grants). Chinese inventors now account for 19% of the top 10% of cited patents considering only patents granted in the period 2015–2019, with the analogous US share of the top 10% patents dropping to under 56.5% in this period. For 2015–2019 patents, there is still a lower Chinese presence in the top 10% of cited AI patents relative to their shares of granted AI patents. However, these results suggest a noticeable rise in the technological importance of more recently granted Chinese AI patents.

**Fig 2:**
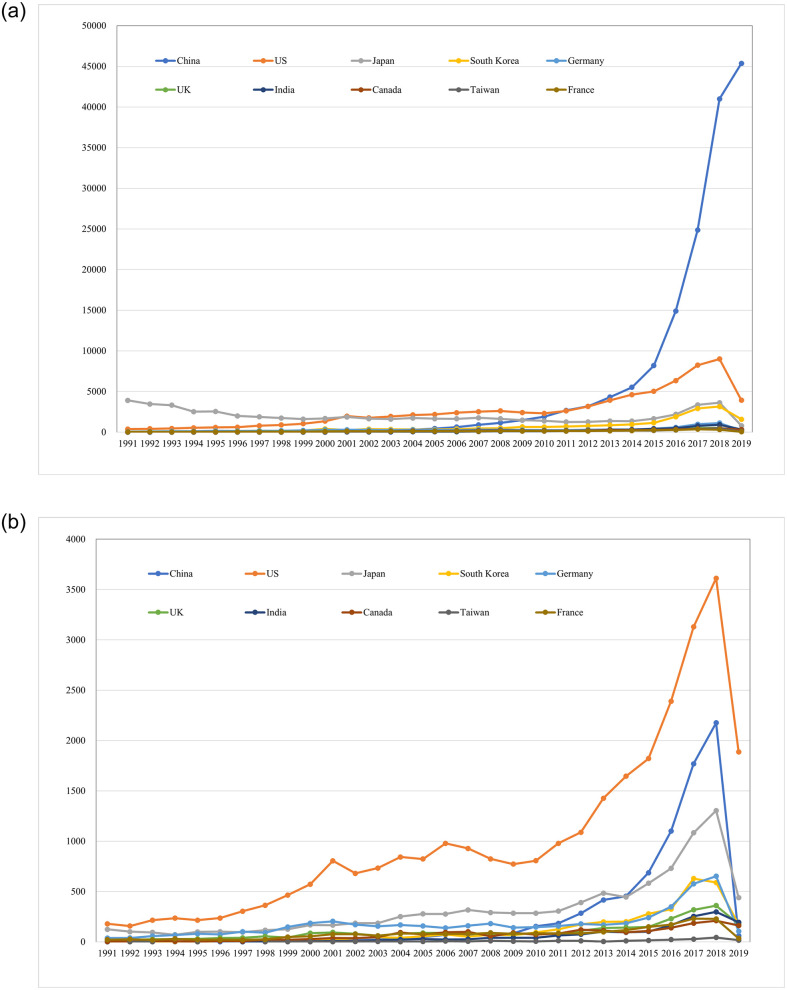
a. AI all patent applications, top 10 countries, 1991–2019. b. AI transnational patent applications, top 10 countries, 1991–2019.

Although Japan was an early first-mover in AI patent applications, it was overtaken by the USA in the early 2000s, then China in 2010 (Fig 2a). Japan’s AI patent applications increased again in the mid-2010s and are now just above those of South Korea. Additionally, while the USA led in AI patent applications for much of the 2000s, China overtook the USA in 2011 and has continued to see a rapid acceleration by quantity of AI patent applications through to 2019 (latest data year). However, the USA has consistently maintained a leading position in transnational patent applications (which potentially signal higher quality inventions) between 1991 and 2019 (Fig 2b). China is less prominent in transnational patent applications, but it has exceeded Germany and Japan since 2015.

#### International co-patenting relationships

**Fig 3:**
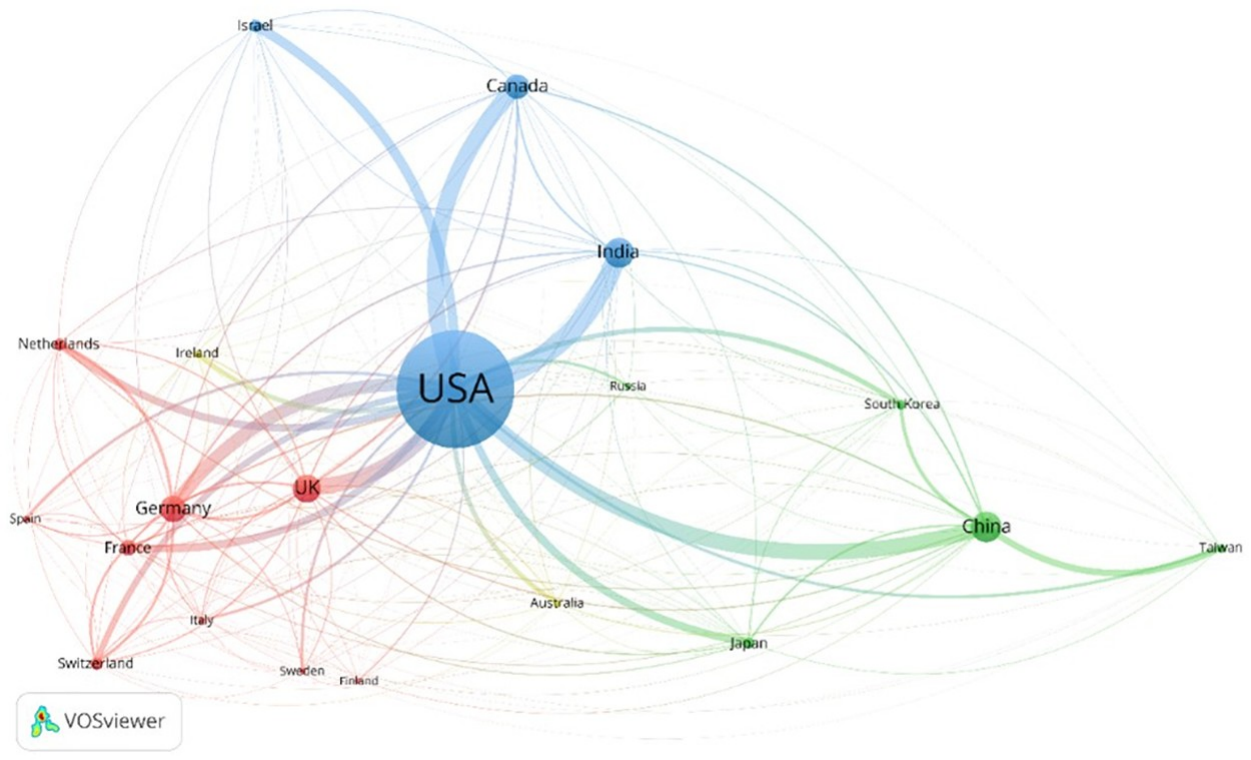
AI co-patenting relationships, top 20 countries (by inventor addresses).

There are about 20,000 patent applications with inventors from multiple countries, accounting only for about 5% of total AI patent applications worldwide (Table 2). This limited level of international cooperation is influenced by China, Japan, and South Korea, which have low rates of international cooperation (2.0%, 1.5% and 5.2%, respectively). The comparable rates for the USA and Taiwan are 19.1% and 21.9%, respectively. The UK, Germany, France, and Canada have higher rates of international co-patenting, with the highest level reached in India (61.7%). However, although its rate of international co-patenting is at a middling level, the USA remains the predominant node in international cooperation due to its absolute count of international cooperation patent applications, accounting for more than 70% of total international cooperation patent applications worldwide. The USA is the hub of the global AI patenting cooperation network and the leading partner for most other countries including China, Canada, India, the UK, and Germany (Fig 3). A European sub-cluster is also evident (with the UK, Germany, and France as key nodes), with China the ley node for a sub-cluster with Taiwan, South Korea, and Japan.

### Organization-level landscape

#### Productive and high-quality organizations

The leading assignees of AI patent applications over the 1991–2019 period are primarily corporations and are concentrated in a relatively small number of countries. Twenty-one of the top 25 AI patent application assignees are companies, led by the USA (3), China (6) and Japan (10), with a further three based in South Korea, Germany, and Taiwan. Four of the top 25 assignees are Chinese universities or institutes. Additionally, all 25 top assignees for transnational patent applications are companies, with 9 for the USA, 7 for Japan, 4 for China, 2 for Germany and each one for South Korea, Netherlands, and Finland, respectively (Table 3). Up to 2004, AI patent applications were concentrated in companies from Japan (led by Canon, NEC, Toshiba, Fujitsu, and Hitachi) and the USA (including IBM, Microsoft, and Alphabet). The top Chinese assignees (including the Chinese Academy of Sciences, the State Grid Corporation, Baidu, Tencent, Ping An Insurance, Alibaba, and Tsinghua, Zhejiang, and Xidian Universities) are more recent entrants, developing AI patent applications particularly from 2005 through to 2019. Samsung (South Korea), Siemens (Germany), and Foxconn (Taiwan) are also consistent AI patent applicants (Table 3).

**Table 3:** Leading assignees, AI patent applications, 1991–2019.

Total patent applications									Transnational patent applications								
Assignee	AOC	1991–2019	1991–94	1995–99	2000–04	2005–09	2010–14	2015–2019	Assignee	AOC	1991–2019	1991–94	1995–99	2000–04	2005–09	2010–14	2015–19
IBM	USA	9444	228	416	896	1265	1969	4670	Microsoft	USA	3175	20	92	371	583	771	1338
Microsoft	USA	6900	47	275	1023	1731	1587	2237	Alphabet	USA	1887	6	30	77	178	466	1130
Chinese Acad Sci	China	4322	4	9	52	191	784	3282	Siemens	Germany	1595	74	162	212	223	216	708
Canon	Japan	4232	931	787	659	690	542	623	Samsung	South Korea	1415	5	7	61	100	361	881
NEC	Japan	4216	1187	655	360	628	590	796	Philips	Netherlands	1270	35	70	150	164	237	614
Toshiba	Japan	4141	1386	925	448	585	384	413	NEC	Japan	1080	12	29	26	214	270	529
State Grid Corp	China	3814	0	1	0	25	506	3282	Sony	Japan	1044	9	83	183	142	143	484
Alphabet	USA	3627	13	92	180	395	1367	1580	IBM	USA	819	133	65	96	118	158	249
Fujitsu	Japan	3483	732	527	387	415	518	904	Nokia	Finland	795	38	101	126	144	170	216
Hitachi	Japan	3424	1109	730	346	312	328	599	Huawei	China	756	0	2	8	47	194	505
Baidu	China	3406	0	0	0	3	299	3104	Intel	USA	737	9	13	56	38	154	467
NTT	Japan	3264	496	489	407	398	650	824	General Electric	USA	627	4	34	97	86	144	262
Samsung	South Korea	3257	39	111	219	396	791	1701	Tencent	China	627	0	0	0	9	220	398
Panasonic	Japan	3213	1302	712	420	194	114	471	Alibaba Group	China	598	0	1	0	11	124	462
Tencent	China	2908	0	0	1	42	350	2515	Panasonic	Japan	597	34	50	85	80	79	269
Ping An Insurance	China	2861	0	0	0	0	1	2860	HP Inc.	USA	581	19	30	92	90	179	171
Fujifilm	Japan	2829	449	394	457	764	340	425	Qualcomm	USA	552	0	8	22	63	253	206
Siemens	Germany	2644	123	258	315	461	463	1024	Hitachi	Japan	545	32	28	33	50	162	240
Alibaba Group	China	2326	1	1	2	19	229	2074	Ping An Insurance	China	543	0	0	0	0	0	543
Foxconn	Taiwan	2273	772	424	276	309	338	154	Fujitsu	Japan	539	19	27	66	81	104	242
Sony	Japan	2232	153	302	463	408	335	571	Mitsubishi Electric	Japan	530	16	57	46	52	117	242
Ricoh	Japan	2120	653	320	435	304	180	228	Canon	Japan	491	74	44	89	95	85	104
Tsinghua Univ	China	1928	1	0	23	79	213	1612	Bosch	Germany	424	10	20	43	70	66	215
Zhejiang Univ	China	1909	6	0	12	139	305	1447	Apple	USA	394	36	17	9	43	112	177
Xidian Univ	China	1710	0	0	0	42	279	1389	Nuance	USA	389	13	80	83	90	91	32

Note: AOC = Assignee original country.

There are 14 companies listed as top assignees for both total and transnational applications, led by Microsoft, Alphabet (the parent company of Google), Siemens, and Samsung, followed by NEC, Sony, IBM, Tencent and Alibaba, which suggests these organizations are both productive and high-quality performers in AI patenting. Eleven organizations are listed only as top assignees for total patent applications (including the Chinese Academy of Sciences, the State Grid Corporation, Baidu, and NTT), while another 11 companies are listed only as top assignees for transnational applications (including Philips, Nokia, Huawei, Intel, General Electric, and Qualcomm) (Table 3).

#### Interorganizational co-patenting and citation networks

**Fig 4:**
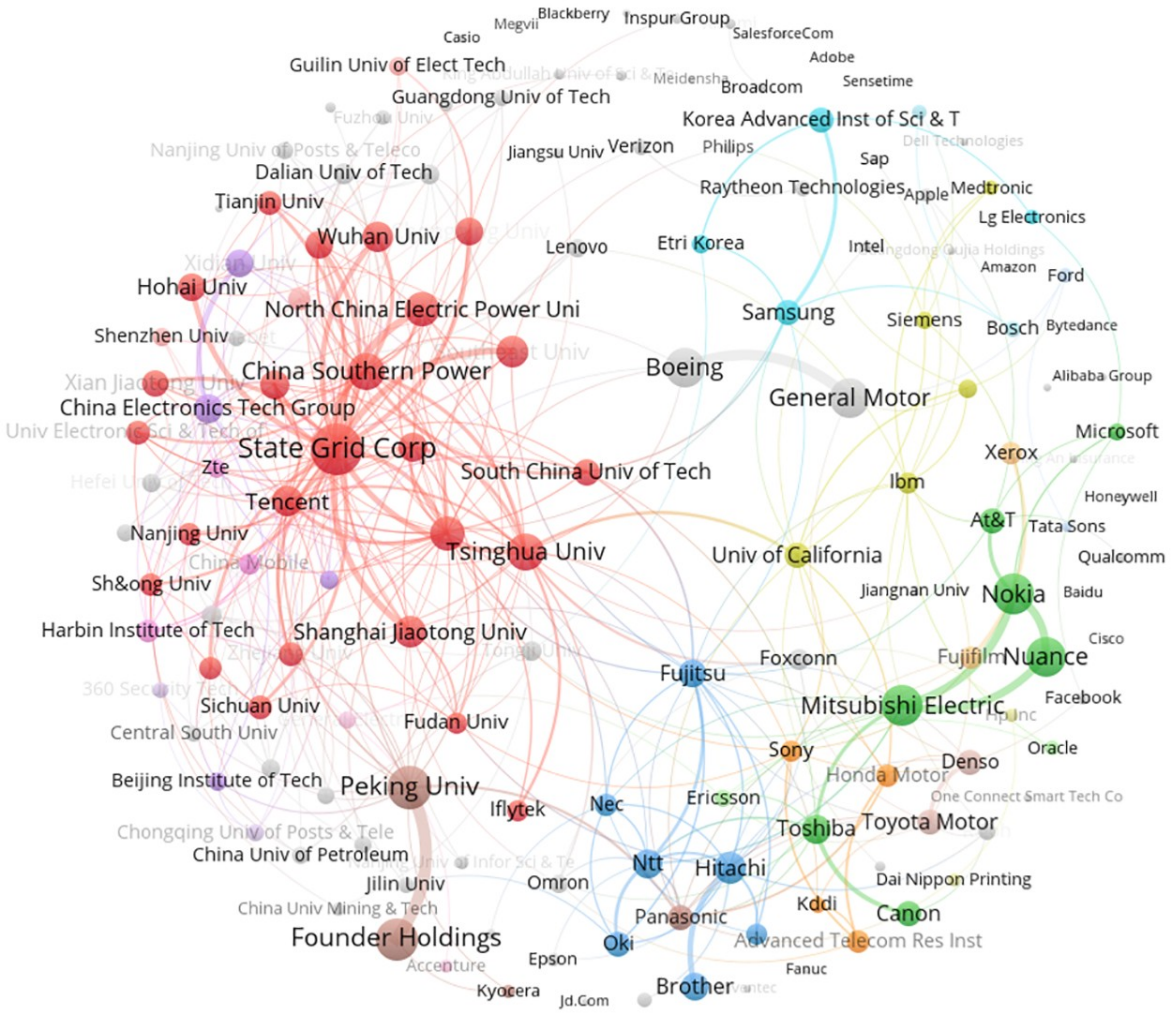
Collaboration networks, top 150 AI patenting applicant organizations, 1991–2019.

At a consolidated level of assignees, organizations mostly filed their AI patent applications as single assignees, with only about 2% of 1991–2019 AI patent applications involving multiple organizations. Yet, the co-patenting network across the 150 most AI patent-intensive organizations is informative (Fig 4). The most obvious co-patenting clusters are among companies and research institutions in China, suggesting interorganizational knowledge flows through social capital [35]. More loosely coupled networks are observable in the USA, South Korea, and Japan.

**Fig 5:**
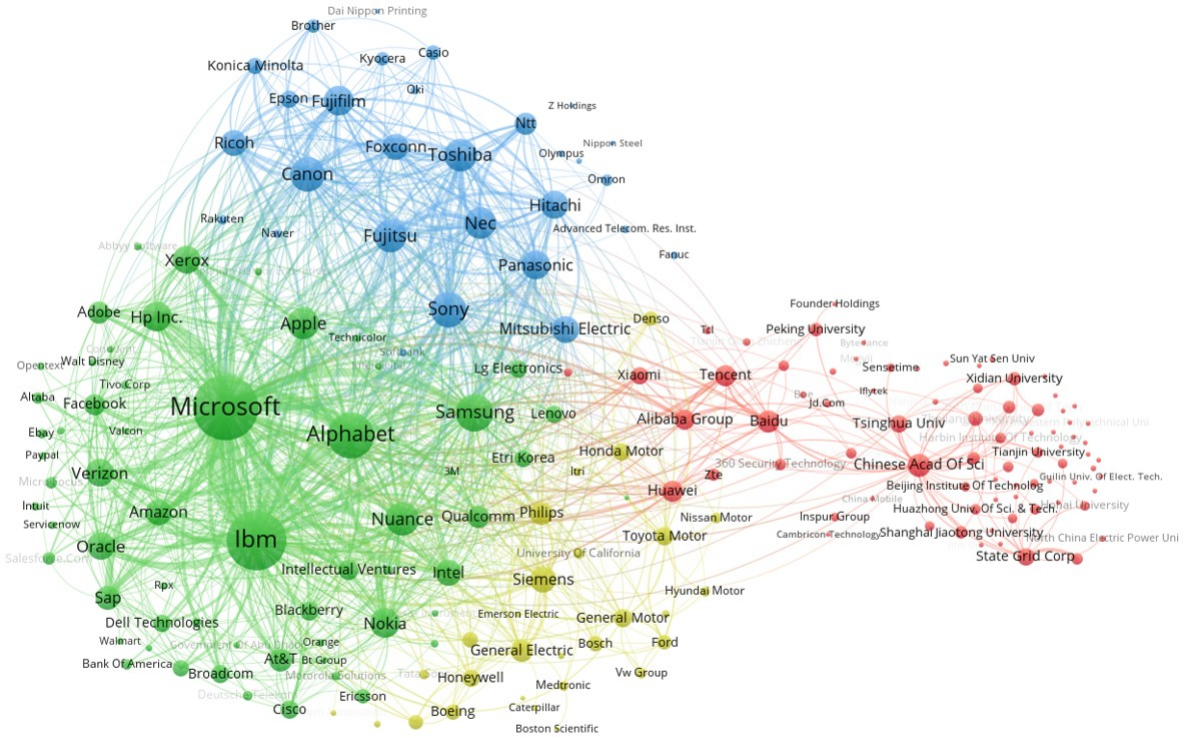
Citation networks, top 150 citing and cited AI patenting organizations, 1991–2019.

Interorganizational knowledge flows can also occur indirectly through citations in patents to other organizations. For AI, such citation relationships are dense, as illustrated in a mapping of links across the top 150 citing and cited organizations (Fig 5). Compared to the dispersed co-patenting network across organizations, this highlights how technological innovation in AI builds upon citation-based rather than collaboration-based knowledge links. We observe several citation clusters. The first cluster (green) mainly involves companies from the USA, including Microsoft, Alphabet, and IBM. At the edges of this cluster are companies from other countries, such as Samsung, Nokia, SAP, Blackberry and Lenovo. The second cluster (blue) involves companies from Japan, such as Sony, Canon, Fujitsu, and Toshiba. The third cluster (red) comprises a dispersed Chinese group including not only firms but also many universities and institutes. Overall, the USA and Japan express an industry-oriented innovation model, while China expresses an industry-university-institute-oriented innovation model in the AI domain.

Based on forward citation relationship among patenting organizations in the AI field, we calculated the technological impacts of organizations worldwide. High-impact organizations mostly originated in the USA (11) and Japan (9), with one each for South Korea, Finland, Germany, China, and Netherlands (Table 4). The higher the technology impact of an organization, the more the organization serves as a knowledge broker. Microsoft, whose technological knowledge for AI has diffused (via forward citations) to more than 5000 organizations, has the highest technology diffusion capacity worldwide (6.2%), followed by IBM, Alphabet and Nuance. IBM, which absorbed technological knowledge (via backwards citations) from more than 1000 organizations, has the highest technology absorptive capacity worldwide (2.3%), followed by Microsoft, Alphabet and Samsung. The weighted PageRank (WPR) for each top organization also indicates their importance in knowledge transmission.

**Table 4:** Top 25 technological high-impact organizations, based on AI patent citations, 1991–2019.

Assignee	AOC	TAI (%)	TDC (%)	TAC (%)	Outdegree	Indegree	WDL	WAL	WPR (%)
Microsoft	US	8.39	6.23	2.17	5157	1052	57201	19904	5.43
IBM	US	7.02	4.40	2.62	4747	1191	40436	24054	4.21
Alphabet	US	4.23	2.58	1.65	3424	939	23711	15171	2.26
Samsung	South Korea	2.77	1.24	1.53	2246	1085	11432	14051	1.00
Sony	Japan	2.37	1.40	0.97	2122	812	12880	8903	1.28
Nuance	US	2.24	1.72	0.53	1918	413	15793	4829	1.66
Canon	Japan	2.11	1.22	0.89	1774	733	11207	8196	1.19
Fujitsu	Japan	1.93	0.98	0.95	1837	840	9035	8693	0.96
Toshiba	Japan	1.90	1.10	0.79	1851	718	10146	7293	1.09
Apple	US	1.88	1.13	0.74	2044	628	10400	6841	1.02
NEC	Japan	1.79	0.98	0.81	1797	718	8981	7467	0.93
Nokia	Finland	1.77	1.09	0.68	1797	679	10045	6236	1.14
HP Inc.	US	1.72	0.96	0.76	1912	712	8826	7018	0.98
Siemens	Germany	1.66	1.01	0.65	2177	860	9254	6012	1.06
Hitachi	Japan	1.62	1.05	0.57	1968	731	9649	5243	1.07
Panasonic	Japan	1.60	1.05	0.55	1850	679	9684	5048	1.03
Oracle	US	1.51	0.90	0.61	1782	566	8263	5598	0.86
Fujifilm	Japan	1.50	0.90	0.60	1535	573	8231	5555	0.88
Xerox	US	1.49	1.05	0.45	1922	529	9641	4090	1.04
Verizon	US	1.47	0.88	0.59	1728	571	8073	5413	0.79
Mitsubishi Electric	Japan	1.43	0.89	0.54	1704	614	8170	4984	0.95
Intel	US	1.41	0.69	0.71	1568	792	6373	6544	0.63
Chinese Acad Sci	China	1.38	0.82	0.56	1485	967	7535	5184	0.45
General Electric	US	1.37	0.84	0.53	1982	771	7742	4852	0.90
Philips	Netherlands	1.32	0.86	0.46	2061	634	7903	4208	0.95

**Note**: AOC = Assignee original country; TAI = Technology absolute impact; TDC = Technology diffusion capacity; TAC = Technology absorptive capacity; WDL = Weighted diffusion links; WAL = Weighted absorptive links; WPR = Weighted PageRank.

**Fig 6:**
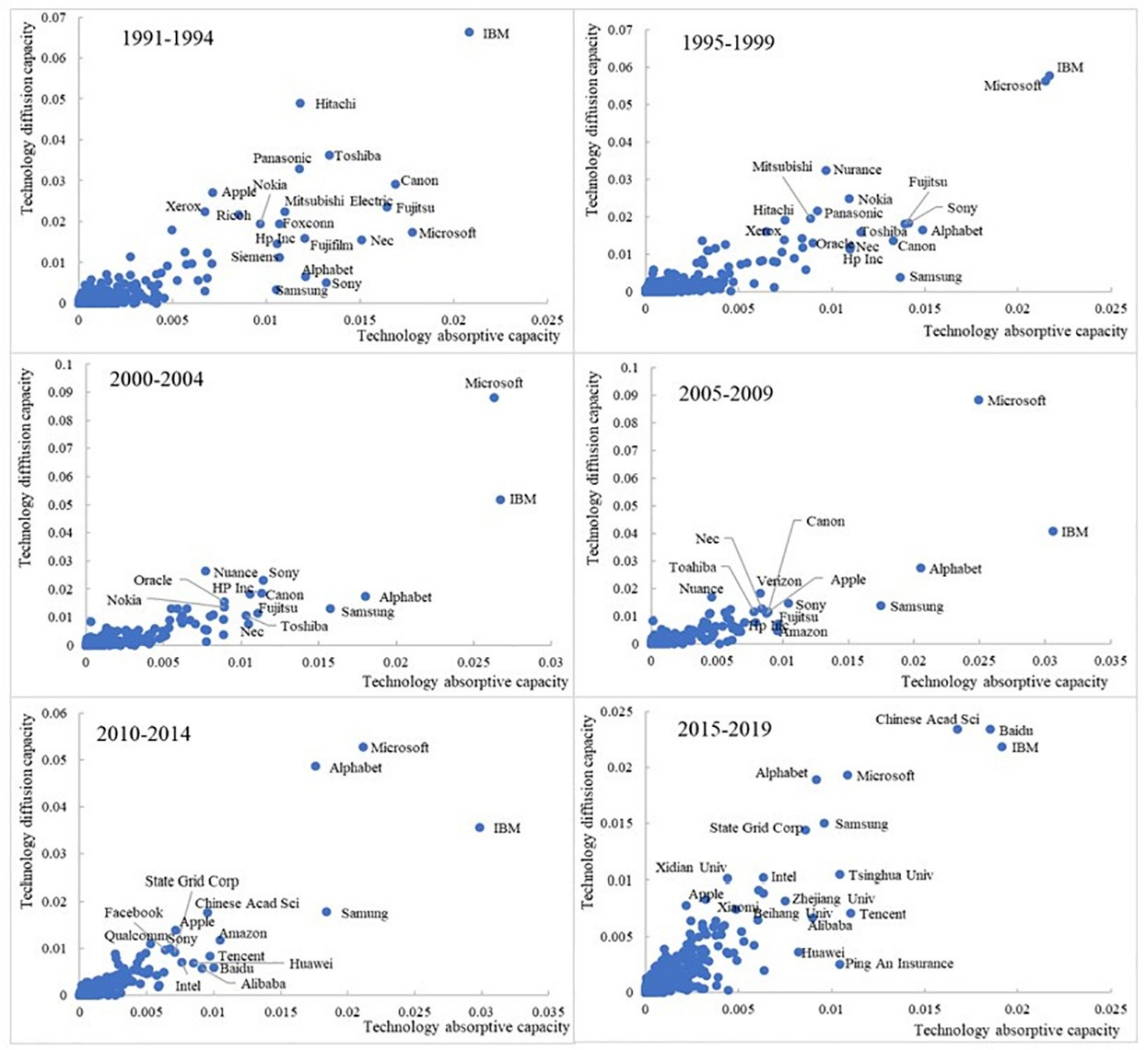
The distribution of technology diffusion capacity and absorptive capacity of organizations.

There is an apparent “Matthew effect” [49] in AI knowledge flows (Fig 6). A few organizations possess high levels of technology diffusion and absorptive capacity; most others are at a lower level in technology impact. Leading US companies, such as Microsoft, IBM, and Alphabet, have maintained a high level of technology impact throughout the 1991–2019 period; the same is true for Samsung (South Korea). Until 2009, Japanese companies, including Sony, Fujitsu, and Toshiba, showed noticeable technology impact, but were less visible from 2010. Conversely, several Chinese organizations have emerged as high-impact organizations since 2010, including the Chinese Academy of Sciences, Tencent, Baidu, Alibaba, and Huawei.

### Technology-level landscape

#### Distribution of technology fields

AI patent applications cover a wide range of technological and application areas. More than 600 IPC 4-digit subclasses are represented in our AI patent data. However, the top 20 IPC subclasses account for over 90% of all AI patent applications, while the more disaggregated top 20 IPC subgroups captured 65% of patent applications (Table 5).

**Table 5:** Top IPCs for AI patent applications, 1991–2019.

IPC subclass	Patent applications					IPC subgroup	Patent applications	
	Count	1991–2019	1991–1999	2000–2009	2010–2019		Count	1991–2019
G06F	173644	48.30%	73.98%	67.70%	40.66%	G06K9/62	43514	12.10%
G06N	89198	24.81%	19.05%	14.41%	27.90%	G06F17/30	43096	11.99%
G06K	68991	19.19%	6.03%	8.66%	23.23%	G06K9/00	33746	9.39%
G06Q	49308	13.71%	5.70%	12.40%	15.01%	G06F17/21	32073	8.92%
G06T	36666	10.20%	8.77%	6.63%	11.19%	G06F17/27	30752	8.55%
G10L	24654	6.86%	8.56%	10.20%	5.88%	G06N3/04	29847	8.30%
H04L	24124	6.71%	2.41%	6.24%	7.35%	G06N3/08	29133	8.10%
A61B	17096	4.75%	1.74%	4.88%	5.10%	G06F17/24	17749	4.94%
H04N	14096	3.92%	3.98%	5.35%	3.59%	G06F17/22	16131	4.49%
G05B	13137	3.65%	5.88%	3.96%	3.31%	G06F17/28	15598	4.34%
G01N	11400	3.17%	2.08%	4.35%	3.04%	G06T7/00	13806	3.84%
G16H	10641	2.96%	0.17%	0.65%	3.84%	G06F19/00	13639	3.79%
G05D	8830	2.46%	1.03%	0.88%	2.99%	G06N99/00	13490	3.75%
H04W	7364	2.05%	0.37%	1.57%	2.37%	G06F17/00	12690	3.53%
G08G	5902	1.64%	0.77%	1.04%	1.89%	G06N5/04	10210	2.84%
H04M	5574	1.55%	1.44%	3.03%	1.23%	G06F15/18	10179	2.83%
G01R	4745	1.32%	0.93%	1.21%	1.39%	G06K9/46	9975	2.77%
B25J	4638	1.29%	0.51%	0.73%	1.51%	G06N5/02	9372	2.61%
G09B	4570	1.27%	1.43%	1.71%	1.15%	A61B5/00	9335	2.60%
G01C	4513	1.26%	0.53%	1.09%	1.39%	H04L29/08	9061	2.52%

**Note**: For explanation of IPC codes, see https://www.wipo.int/classifications/ipc/en/ and S1 Appendix. AI patent search approach.

The largest AI patenting class is “computing, calculating, or counting” (G06) classifying almost 78% of all AI patent applications (1991–2019). At the subclass level, nearly a half of AI patents are classified under “electric digital data processing” (G06F), although with a decline from about 70% of AI patent applications in 1991–1999 to about 40% in 2010–2019. This technology subclass is followed by “computer systems based on specific computational models” (G06N, 24.8%), “recognition and presentation of data” (G06K, 19.2%), “data processing systems or methods for special purposes” (G06Q, 13.7%) and “image data processing or generation” (G06T, 10.2%), whose relative shares increased significantly over time (Table 5). These classifications are fundamental AI technologies associated with improvements in computing capabilities.

For specific subgroups of AI technologies, “methods or arrangements for recognition using electronic means” (G06K 9/62) ranks first with 12.1% of all patent applications. “Information retrieval; Database structures therefor” (G06F 17/30) ranks second with 12.9% of AI patent applications. “Recognition patterns” (G06K 9/00) ranks third at 9.4%. There are several top IPC subgroups related to “handling natural language data, including text processing” (G06F 17/21, 8.92%; G06F 17/24, 4.94%; G06F 17/22, 4.49%), “automatic analysis” (G06F 17/27, 8.55%), “processing or translating of natural language” (G06F 17/28, 4.34%). “Architecture” (G06N 3/04) and “learning methods” (G06N 3/08) related to “using neural network models” account for 8.3% and 8.1% of AI patent applications, respectively (Table 5).

#### Key technological development routes

**Fig 7:**
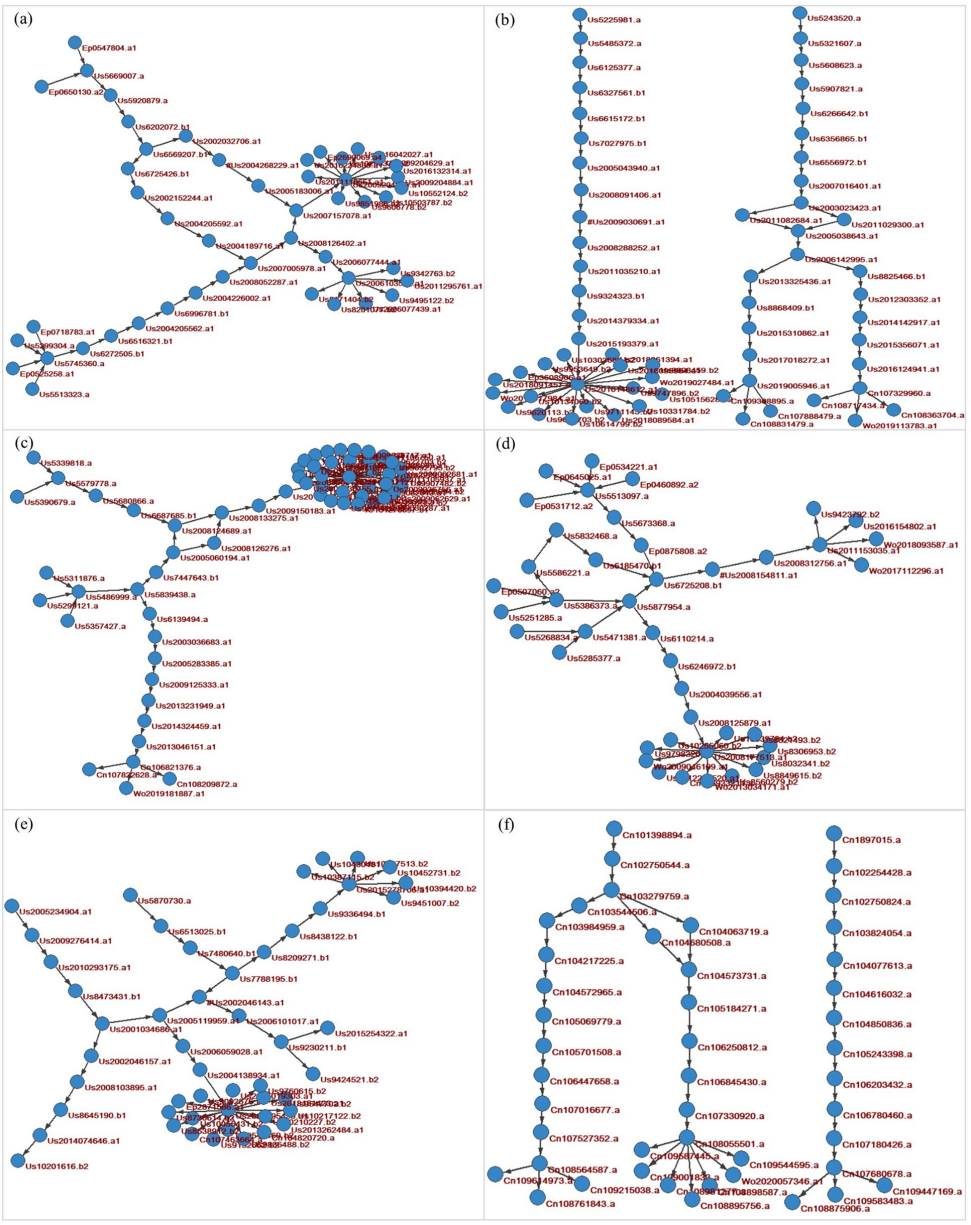
Key technology routes for AI patents.

This section analyzes the main technological trajectories of AI technologies through extracting key technological routes from citation networks [50]. We use the Louvain method for community detection [51] available in the Pajek software package [52]. We extracted the key technological routes using the SPLC algorithm from the subnets of the six largest communities of citation networks for AI technologies. These six communities represent 41% of all nodes and 45% of all arcs in the citation network for AI technologies. The results are shown in Fig 7a–7f, where nodes refer to patents and arcs refer to links obtained through forward patent citations, indicating knowledge flow between patents.

We analyze the first application country and priority authorities of patents in these technological routes. For four technological routes (Fig 7a–7c and 7e), most patents are applied and prioritized in the USA, with 79%, 76%, 90% and 74%, respectively. Japan is also a leading country in technological routes shown in Fig 7a, with 14% prioritized patents. China is significant in the technological route shown in Fig 7b, with 12% prioritized. Russia is significant in the technological route shown in Fig 7e, with 10% prioritized patents. The technological route shown in Fig 7d is jointly dominated by the USA, Germany, and Japan, respectively with 57%, 21% and 14% patents applied and prioritized. The technology routes shown in Fig 7a–7e are industry-dominated, with 73%-94% of all patents assigned to firms. This contrasts with the university-institute-industry-oriented technological route depicted in Fig 7f, where all patents are applied and prioritized in China, with 43% of patents assigned to firms and 48% assigned to universities or research institutes.

Analyzing interorganizational co-ownership for patents involved in key technological routes, we find that Fig 7a has no patents with co-ownership across organizations, Fig 7b has only five patents with co-ownership across organizations, and Fig 7c–7f each have just one patent of this type. In other words, for AI technological routes, there is a preference by organizations for exclusive technology development.

By technology topics (analyzed by IPC codes and keywords in patent abstracts), Fig 7a mainly covers technologies handling natural language data (G06F 17/20, G06F 40/00), specifically including text processing (G06F 17/21, G06F 17/22, G06F 17/24) and automatic analysis (G06F 17/27). Patents in the technological routes shown in Fig 7b are mainly classified into handling natural language data (G06F 17/27, G06F 17/28), information query (G06F 17/30) and speech recognition (G10L 15/02, G10L 15/06, G10L 15/14, G10L 15/18, G10L 15/22, G10L 15/24, G10L15/26, G10L 15/28). Patents in the technological route shown in Fig 7c mainly target technologies of digital computing or data processing equipment or methods that are specially adapted for specific applications (G06F 19/00) or for specific functions (G06F 17/00) and measuring for diagnostic purposes (A61B 5/00, A61B 5/0476). The technological route shown in Fig 7d involves technologies about adaptive control systems (G05B 13/02, G05B 13/04) and learning machines (G06F 15/18) such as sensors and controllers, which basically use biological models (G06N 3/00), especially neural network modeling (G06N 3/02, G06N 3/04). Patents in the technological route shown in Fig 7e cover technologies of machine learning (G06F 15/18, G06N 20/00), information retrieval and data structures (G06F 17/30), knowledge representation (G06N 5/02) and inference methods or devices (G06N 5/04). This technological route addresses problems of prediction, ranking and recommendation involved in administration, management, business, or financial fields by using machine learning technologies. Patents in the technological routes shown in Fig 7f are mainly classified into recognizing patterns (G06K 9/00, G06K 9/20, G06K 9/46, G06K 9/60, G06K 9/62, G06K 9/78), image analysis (G06T 7/00, G06T 7/11, G06T 7/12, G06T 7/13, G06T 7/33, G06T 7/246, G06T 7/73) and using neural network models (G06N 3/08, G06N 3/04, G06N 3/02).

## Discussions and conclusions

In this study, we put forward an approach to identify AI patents and applied this to offer a broad global analysis of the AI patenting landscape at country, organization, and technology levels. We discussed the details of the methodological approach to operationalize a new search approach which captures AI-related patents with recall and precision. To assess patent productivity and quality, we examined total patent applications and transnational patent applications respectively, using cross-sectional and longitudinal views. This analysis highlighted AI patent developments in leading countries and organizations. The study also examined co-patenting collaborations and forward citation relationships, to understanding knowledge sharing among organizations. Based on forward citation relationships among organizations, we analyzed technological knowledge inflows and outflows in interorganizational citation networks and identified patent-based technological routes to identify development trajectories and core technologies in the AI domain.

Our analysis of patent application data over two decades (1991–2020) finds that activity in AI patenting is currently in a phase of rapid growth, driven significantly (although not exclusively) by a recent increase of AI patenting in China. From a geographical perspective, AI patenting efforts remain highly concentrated, with inventors in the top 10 countries contributing almost 96% of all worldwide AI patent applications. The USA, China and Japan are among the most patent-intensive and specialized countries in the AI domain, followed by South Korea, Germany, and the UK. Over the past three decades, the USA has maintained a leading position, with China growing most rapidly in recent years and Japan seeing relative loss of its early leadership position.

The analysis of top assignees showed that most AI patents are owned by large private companies. Public organizations and universities are less prominent in the ownership of AI patent applications, except in China where public research organization and universities are frequent AI patent assignees. In general, the USA and Japan each exhibit an industry-oriented innovation model, while China expresses an industry-university-institute-oriented innovation model in AI patent development. AI co-patenting collaborations through joint ownership are not common. However, there are many interorganizational knowledge citation relationships, although they are geographically bounded. Matthew effects of accumulated advantage are apparent, with a few organizations acting as high-impact technology disseminators and acceptors.

We demonstrated that AI patents cover a wide range of technological areas, not only in information technologies and computing but also in applied fields such as medicine, healthcare, finance, and education. The main path analysis highlighted multiple AI technological development paths. However, we found that flows of technological knowledge tend to be concentrated among patents developed in the same country, mostly dominated by the USA although there were some technological routes led by China. This analysis confirmed the strong presence of independent assignees and a lack of collaborative patent assignments among organizations in the AI domain.

There are a series of implications that can be drawn from the study for management and policy. AI is emerging as a general-purpose technology with applications, as we have shown, across multiple fields. The current boom in AI patenting reminds us of the need for continuing attention to the challenges of AI applications, including societal consequences. For managers and analysts in all sectors, our study highlights the strategic relevance of tracking (if not engaging in) AI-enabled developments, opportunities, and competitive challenges. A way to do this is through monitoring rapidly increasing efforts to secure intellectual property in AI through patent applications. Our search approach can be used (and further modified or refined) to assist monitoring of AI patents. Patent applications do vary in quality, as we have shown, and not all patent applications are granted. Nonetheless, this growing body of codified information (especially if searched appropriately) provides a basis for tracking developments and furthering AI knowledge progression and innovation as potential approaches and processes are disclosed in published patent applications. Inventors and organizations in the USA, Japan and selected other developed countries continue to be well-represented in high quality AI patenting activity, but we also find that AI patenting efforts in China are growing not only in terms of patent quantity but are also (especially recently) increasing in patent quality.

While AI patenting trends by countries, organizations, and technologies are of interest to policymakers, our findings about the dominant roles of a subset of leading multi-national organizations in AI patenting also raise ongoing policy questions about the control and management of emerging AI technologies and applications. Indeed, the observed concentration of AI patenting in a few countries and organizations raises multiple policy issues, including how to moderate information and power asymmetries between developers and users of the technologies and between incumbents and new entrants. Additionally, the current weaknesses of collaborative development highlighted in AI patenting may cause problems in technological and market reach and for standardization and interoperability.

We acknowledge limitations that should be kept in in mind when interpreting the results of the study. As we have discussed, although patent data is widely used as a proxy for innovation, it has shortcomings. To avoid disclosure, some firms may choose not to patent their technological innovations but to hold them as business secrets. Although patent application data do signal inventive trends and assignee interests, it should be noted that not all patent applications are granted, nor are granted patents necessarily used, maintained, or enforced. Our research has taken a broad landscape perspective. Further research is needed to pinpoint trends in specific sub-technological AI fields and to probe implications by specific application areas.

## Supporting information

S1 Appendix AI patent search approach. Summary of bibliometric search term method and selection of AI-related CPC and IPC patent codes.
(PDF)
