# Supplementary material for: Mapping technological innovation dynamics in artificial intelligence domains: Evidence from a global patent analysis
Source: PLoS One. 2021 Dec 31;16(12):e0262050. doi: 10.1371/journal.pone.0262050 (PMC8719762; doi:10.1371/journal.pone.0262050)
Supplement: S1 Appendix — Summary of bibliometric search term method and selection of AI-related CPC and IPC patent codes. (PDF) [file pone.0262050.s001.pdf]

Appendix S1. Supporting Information

AI patent search approach

The artificial intelligence (AI) patent search approach used in the paper combines a keyword-based search with International Patent Classification (IPC) and Cooperative Patent Classification (CPC) codes to select AI-related patents. The AI keywords and selected CPC and IPC codes are reported in the paper (Table 1, see row #1 for keywords and rows #2 and #3 respectively for the selected IPC and CPC codes), with added information on the methods used for selection summarized below. A patent document is identified as AI-related if its title, abstract or claims match at least one AI keyword, or it is assigned at least one of the CPC or IPC AI codes (Table 1, row #4).

Summary of bibliometric search term method

The AI-related key word search uses the terms identified through our previously published bibliometric approach (see: Liu et al., 2021. This provides full details of the search method and is available open access at https://doi.org/10.1007/s11192-021-03868-4). In summary, this approach proceeded through three stages.

- We used the core lexical query "artificial intelligence" as a topic search and a query of specialized AI journals as a source search to generate a benchmark set of AI publications from the Web of Science.
- From these benchmark AI records, we extracted "Author Keywords" and "Keywords Plus" and derived the frequencies of these keywords. We confirmed the precise meanings of high-frequency keywords from descriptions found in online sources. This process led to a retained list of high-frequency "candidate keywords" related to AI.
- To maintain balance between recall and precision, we test and refine this set of terms through co-occurrence analysis and manual checking identification. Nine keywords that frequently co-occurred with the central term "artificial intelligence" are added as part of core lexical query. For the remaining candidate keywords, we introduced a "Hit Ratio" metric and performed manual checking, excluding candidate keywords from our final search list if deemed to have a high noise ratio

Testing of this approach indicated relatively high recall and precision in capturing AI-related document records.

Selection of AI-related CPC and IPC patent codes

We manually selected AI-related CPC (Cooperative Patent Classification, https://www.cooperativepatentclassification.org/) and IPC (International Patent Classification, https://www.wipo.int/classifications/ipc/en/) codes to supplement the search results of AI patents captured by AI-related search terms. Each of the selected CPC and IPC codes was tested using the PatentSight database, with random (100 record) samples of search results manually checked to assess the recall and precision. If greater than 70% of a sample comprised patents relevant to artificial intelligence, the focal CPC or IPC code was included in our final search strategy, with this patent code deemed as having a low noise ratio. (See Table 1, rows #2 and #3, and Table S1 for text descriptions for selected codes.)

Table S1. Descriptions for AI-specific CPC and IPC codes

CPC/IPC Codes	Description
A61B 5/7264	...{Classification of physiological signals or data, e.g. using neural networks, statistical classifiers, expert systems or fuzzy systems (neural networks per se G01N3/00; expert systems per se G06N5/00)}
A61B 5/7267	.... {involving training the classification device}
A63F 13/67	adaptively or by learning from player actions, e.g. skill level adjustment or by storing successful combat sequences for re-use
B23K 31/006	. {relating to using of neural networks}
B25J 9/161	... {Hardware, e.g. neural networks, fuzzy logic, interfaces, processor}
B25J 9/163	learning, adaptive, model based, rule based expert control
B29C 66/965	... {using artificial neural networks}
B29C 2945/76946	using an expert system, i.e. the system possesses a database in which human experience is stored, e.g. to help interfering the possible cause of a fault
B29C 2945/76949	using a learning system, i.e. the system accumulates experience from previous occurrences, e.g. adaptive control
B29C 2945/76979	... Using a neural network
B60G 2600/1876	...Artificial intelligence
B60G 2600/1878	... Neural Networks
B60L 2260/46	by self learning
B60T 8/174	.. characterised by using special control logic, e.g. fuzzy logic {, neural computing}
B60T 2210/122	... using fuzzy logic, neural computing
B64G 2001/247	... {Advanced control concepts for autonomous, robotic spacecraft, e.g. by using artificial intelligence, neural networks or autonomous agents}
B65H 2557/38	.. for neural adaptive control
B66B 7/043	using learning
B66B 7/045	: using learning
E21B 2041/0028	. {Fuzzy logic, artificial intelligence, neural networks, or the like}
F01N 2900/0402	using adaptive learning
F02D 41/1405	.... {Neural network control}
F03D 7/046	..... {with learning or adaptive control, e.g. self-tuning, fuzzy logic or neural network}
F05B 2270/709	. Type of control algorithm---.. with neural networks
F05D 2270/709	. Type of control algorithm---.. with neural networks
F16H2059/086	Adaptive mode, e.g. learning from the driver
F16H 2061/0084	. {characterised by a particular control method}---.. {Neural networks}
F16H2061/0087	Adaptive control, e.g. the control parameters adapted by learning
G01N 29/4481	. Processing the detected response signal {, e.g. electronic circuits specially adapted therefor (digital signal processing per se G06F17/00)}---.. {Neural networks}
G01N 30/8662	Expert systems; optimising a large number of parameters
G01N 33/0034	..... {comprising neural networks or related mathematical techniques}

G01N 2201/1296	... using neural networks
G01R 31/2846	.... {using hard- or software simulation or using knowledge-based systems, e.g. expert systems, artificial intelligence or interactive algorithms}
G01R 31/3651	... {comprising digital calculation means, e.g. for performing an algorithm}---.... {Software aspects, e.g. battery modelling, using look-up tables, neural networks}
G01S 7/417	... {involving the use of neural networks}
G05B 13/027	... {using neural networks only}
G05B 13/028	: using expert systems only
G05B 13/0285	... {using neural networks and fuzzy logic}
G05B 13/029	... {using neural networks and expert systems}
G05B 13/0295	... {using fuzzy logic and expert systems}
G05B 23/0229	knowledge based, e.g. expert systems; genetic algorithms
G05B 23/024	..... {Quantitative history assessment, e.g. mathematical relationships between available data; Functions therefor; Principal component analysis [PCA]; Partial least square [PLS]; Statistical classifiers, e.g. Bayesian networks, linear regression or correlation analysis; Neural networks}
G05B 23/0254	..... {based on a quantitative model, e.g. mathematical relationships between inputs and outputs; functions: observer, Kalman filter, residual calculation, Neural Networks}
G05B 23/0281	..... {Quantitative, e.g. mathematical distance; Clustering; Neural networks; Statistical analysis}
G05B2219/13111	Expert system
G05B2219/13166	Program intelligent I-O separate from main plc
G05B 2219/21002	... Neural classifier for inputs, groups inputs into classes
G05B2219/23253	Expert system
G05B2219/23288	Adaptive states | learning transitions
G05B2219/24086	Expert system, guidance operator, locate fault and indicate how to repair
G05B 2219/25255	.. Pc structure of the system---... Neural network
G05B2219/31351	Expert system to select best suited machining centre
G05B2219/31352	Expert system integrates knowledges to control workshop
G05B2219/31353	Expert system to design cellular manufacturing systems
G05B2219/31354	Hybrid expert, knowledge based system combined with ann
G05B 2219/32193	... Ann, neural base quality management
G05B2219/32327	Structure, fuzzy logic expert system scheduler
G05B 2219/32329	Real time learning scheduler, use ann logic
G05B 2219/32334	use of reinforcement learning, agent acts, receives rewards
G05B 2219/32335	... Use of ann, neural network
G05B 2219/33002	Artificial intelligence AI, expert, knowledge, rule based system KBS
G05B 2219/33013	... Higher order multilayer artificial neural network ANN, input terms has square, cubic terms of input, output
G05B 2219/33014	... BAM bidirectional associative memory artificial neural network
G05B 2219/33015	time delay artificial neural network
G05B 2219/33021	... Connect plural macrocircuits, neural network modules in a larger network
G05B 2219/33024	... RAM artificial neural network, several lookup tables addressed by input section, output summed
G05B 2219/33025	... Recurrent artificial neural network
G05B 2219/33026	Wavelet artificial intelligence, wavelet orthogonal decomposition for artificial neural network approximation in
G05B 2219/33027	... Artificial neural network controller
G05B 2219/33028	Function, rbf radial basis function network, gaussian network
G05B 2219/33029	... ANNS artificial neural network with sigmoid function
G05B 2219/33033	... Identification neural controller copies weight to system neural controller
G05B2219/33034	Online learning, training
G05B 2219/33035	... Slow learning combined with fast learning artificial neural network, two time scale ann
G05B 2219/33038	: Real time online learning, training, dynamic network
G05B 2219/33039	... Learn for different measurement types, create for each a neural net
G05B 2219/33041	... Structure optimization and learning of artificial neural network by genetic algorithm
G05B 2219/33044	... Supervised learning with second artificial neural network
G05B 2219/33056	Reinforcement learning, agent acts, receives reward, emotion, action selective
G05B2219/33065	Ontogenetic learning, agent learns and adapt its own behaviour
G05B2219/33066	Phylogenetic learning, group agents learn and adapts their behaviour
G05B2219/33295	Fuzzy expert system for diagnostic, monitoring
G05B2219/33303	Expert system for diagnostic, monitoring use of tree and probability
G05B 2219/33321	Observation learning
G05B2219/33322	Failure driven learning
G05B 2219/34066	... Fuzzy neural, neuro fuzzy network
G05B 2219/34081	Fuzzy art map neural network, one art for input map, lookup table, other for output

G05B 2219/34082	Learning, online reinforcement learning
G05B2219/36039	Learning task dynamics, process
G05B2219/36456	Learning tool holding dynamics
G05B 2219/39071	Solve inverse kinematics by ann learning nonlinear mappings, consider smoothness
G05B2219/39072	Solve inverse kinematics by linear hopfield network
G05B 2219/39095	Use neural geometric modeler, overlapping spheres
G05B 2219/39268	Layer perceptron, drive torque from state variables
G05B 2219/39271	Ann artificial neural network,ffw-nn, feedforward neural network in
G05B2219/39276	FFW and PD and ANN for compensation position error
G05B2219/39282	FFW ann for torque command, adapt as function of speed and detected speed
G05B2219/39283	Ffw ann to compensate torque or speed
G05B 2219/39284	.. Robotics, robotics to robotics hand----... NSC neural servo controller
G05B 2219/39286	... Forward inverse, dynamics model, relaxation neural network model firm
G05B 2219/39292	... Neural brain based controller based on simplified model of vertebrate nervous system
G05B2219/39294	Learn inverse dynamics, ffw decomposed ann adapted by pid
G05B 2219/39297	First learn inverse model, then fine tune with ffw error learning
G05B 2219/39298	Trajectory learning
G05B 2219/39311	multilayer, mnn, four layer perceptron,sigmodial neural network in
G05B 2219/39312	double neural network for tracking ,slave mciroprocessor for servo control
G05B 2219/39352	Feedback error learning, ffw ann compensates torque, feedback from pd to ann
G05B2219/39372	Expert rule based system to correct parameters impedance controller
G05B2219/39374	Ffw and ann combined to compensate torque
G05B2219/39376	Hierarchical, learning, recognition and skill level and adaptation servo level
G05B 2219/39385	... Hybrid control system with neural brain based controller and classical ctrler
G05B 2219/40107	Offline task learning knowledge base, static planner controls dynamic online
G05B2219/40115	Translate goal to task program, use of expert system
G05B2219/40408	Intention learning
G05B 2219/40494	neural network for object trajectory prediction, fuzzy for robot path in
G05B2219/40496	Hierarchical, learning, recognition level controls adaptation, servo level
G05B 2219/40499	Reinforcement learning algorithm
G05B 2219/40528	Ann for learning robot contact surface shape
G05B 2219/40529	neural network based on distance between patterns in
G05B 2219/41054	... Using neural network techniques
G05B2219/42018	Pid learning controller, gains adapted as function of previous error
G05B2219/42135	Fuzzy model reference learning controller, synthesis, tune rule base automatically
G05B2219/42141	Filter error learning
G05B2219/42142	Fuzzy control learning of starting friction coefficient
G05B2219/42149	During learning relation between control and controlled signal, open loop
G05B2219/42287	On feedback failure, use profile stored in memory during learning
G05B2219/49065	Execute learning mode first for determining adaptive control parameters
G05D 1/0088	.{characterized by the autonomous decision making process, e.g. artificial intelligence, predefined behaviours (using knowledge based models G06N5/00)}
G05D 1/0221	....{involving a learning process}
G06F 7/023	adaptive, e.g. self learning
G06F 11/1476	... {in neural networks}
G06F 11/2257	.. {using expert systems}
G06F 11/2263	.. {using neural networks}
G06F 15/18	in which a program is changed according to experience gained by the computer itself during a complete run|learning machines adaptive control systems G05B 13/00; artificial intelligence G06N in：
G06F 16/243	Natural language query formulation
G06F 16/24522	Translation of natural language queries to structured queries
G06F 16/3329	Natural language query formulation or dialogue systems
G06F 16/3344	using natural language analysis
G06F 16/90332	Natural language query formulation or dialogue systems
G06F 17/20	handling natural language data speech analysis or synthesis G10L
G06F 17/2282	Automatic learning of transformation rules, e.g. by example in:
G06F 17/28	Processing or translating of natural language G06F 17/27 takes precedence
G06F 17/2881	natural language generation
G06F 17/289	Use of machine translation, e.g. multi-lingual retrieval, server side translation for client devices, real-time translation data retrieval G06F 16/00, administrative and business methods G06Q 10/00, G06Q30/00

G06F 17/30401	Natural language query formulation natural language analysis, translation, semantics G06F17/27, G06F 17/28
G06F 17/3043	Translation of natural language queries to structured queries natural language analysis, translation, semantics G06F 17/27, G06F 17/28
G06F 17/30654	Natural language query formulation or dialogue systems
G06F 17/30684	using natural language analysis
G06F 17/30976	Natural language query formulation or dialogue systems
G06F 19/24	for machine learning, data mining or biostatistics, e.g., pattern finding, knowledge discovery, rule extraction, correlation, clustering or classification
G06F 19/345	medical expert systems, neural networks or other automated diagnosis computer systems utilizing knowledge based models G06N5/00; neural networks per se G06N3/02
G06F 19/707	using machine learning, data mining or chemometrics, e.g., pattern recognition, knowledge discovery, rule extraction, correlation, clustering or classification, chemical name to structure conversion use of machine learning, data mining or biostatistics for processing genetic or protein-related data G06F 19/24
G06F 2207/4824	..... Neural networks
G06K 7/1482	..... {using fuzzy logic or natural solvers, such as neural networks, genetic algorithms and simulated annealing}
G06K 9/6256	Obtaining sets of training patterns; Bootstrap methods, e.g. bagging, boosting
G06K 9/6264	the supervisor being an automated "intelligent" module, e.g. "intelligent oracle"
G06K 9/6269	.... {based on the distance between the decision surface and training patterns lying on the boundary of the class cluster, e.g. support vector machines}
G06K 9/627	based on distances between the pattern to be recognised and training or reference patterns
G06K 9/6273	Smoothing the distance, e.g. Radial Basis Function Networks
G06N 3/004	. {Artificial life, i.e. computers simulating life}
G06N 3/008	.. {based on physical entities controlled by simulated intelligence so as to replicate intelligent life forms, e.g. robots replicating pets or humans in their appearance or behaviour}
G06N 3/02	. using neural network models (for adaptive control G05B13/00; for image pattern matching G06K9/00; for image data processing G06T1/20; for phonetic pattern matching G10L15/16)
G06N 3/0427	in combination with an expert system
G06N 3/0445	Feedback networks, e.g. hopfield nets, associative networks
G06N 3/0463	Neocognitrons
G06N 3/0481	Non-linear activation functions, e.g. sigmoids, thresholds
G06N 3/049	Temporal neural nets, e.g. delay elements, oscillating neurons, pulsed inputs
G06N 3/06	Physical realisation, i.e. hardware implementation of neural networks, neurons or parts of neurons
G06N 3/08	.. Learning methods
G06N 3/084	: Back-propagation
G06N 3/086	using evolutionary programming, e.g., genetic algorithm
G06N 5	: Computer systems using knowledge-based models
G06N 5/00	: Computer systems using knowledge-based models (Not otherwise classified)
G06N 5/02	: Knowledge representation
G06N 5/043	: Distributed expert systems; Blackboards
G06N 7/023	: Learning or tuning the parameters of a fuzzy system
G06N 7/046	... {Implementation by means of a neural network (neural networks using fuzzy logic G06N3/0436)}
G06N 20	Machine learning
G06N 20/00	Machine learning(NOT otherwise classified)
G06N 20/10	using kernel methods, e.g. support vector machines [SVM]
G06N 20/20	Ensemble learning
G06N 99/005	Learning machines, i.e. computer in which a programme is changed according to experience gained by the machine itself during a complete run neural networks G06N3/02; knowledge based models G06N5/00; fuzzy logic systems G06N/02; adaptive control systems G05B13/00
G06T 3/4046	. .{using neural networks}
G06T 9/002	. {using neural networks}
G06T 2207/20081	..Training; Learning
G06T 2207/20084	..Artificial neural networks [ANN]
G07C2009/00849	programming by learning
G07C2009/00888	programming by learning
G07D 7/2083	Learning
G08B 29/186	... {Fuzzy logic; neural networks}
G08G 1/096888	where input information is obtained using learning systems, e.g. history databases
G10H 2250/311	. Neural networks for electrophonic musical instruments or musical processing, e.g. for musical recognition or control, automatic composition or improvisation (musical analysis G10H2210/031; neural networks G06N3/02)

G10K 2210/3024	...Expert systems, e.g. artificial intelligence
G10K 2210/3038	...Neural networks
G10L 15/06	Creation of reference templates | Training of speech recognition systems, e.g. adaptation to the characteristics of the speaker's voice G10L15/14 takes precedence
G10L 15/144	Training of HMMs
G10L 15/16	.. using artificial neural networks
G10L 15/18	using natural language modelling
G10L 17/18	artificial neural networks|connectionist approaches
G10L 25/30	.. using neural networks
G11B 20/10518	... {optimisation methods}----.... {using neural networks}
G16B 40	: ICT specially adapted for biostatistics | ICT specially adapted for bioinformatics-related machine learning or data mining, e.g. knowledge discovery or pattern finding
G16C 20/70	Machine learning, data mining or chemometrics
G16H 50/20	for computer-aided diagnosis, e.g. based on medical expert systems
G21D 3/007	Expert systems
G21D2003/007	Expert systems
H01H2009/566	with self learning, e.g. measured delay is used in later actuations
H01H2047/009	with self learning features, e.g. measuring the attracting current for a relay and memorising it
H01J 2237/30427	....using neural networks or fuzzy logic
H01M 8/04992	...characterised by the implementation of mathematical or computational algorithms, e.g. feedback control loops, fuzzy logic, neural networks or artificial intelligence
H02H 1/0092	. {concerning the data processing means, e.g. expert systems, neural networks}
H02P 21/0014	.. {using neural networks}
H02P 21/0025	implementing a off line learning phase to determine and store useful data for on-line control
H02P 23/0018	.. {using neural networks}
H02P 23/0031	implementing a off line learning phase to determine and store useful data for on-line control
H03H 2017/0208	... {using neural networks}
H03H 2222/04	. using neural networks
H04L 12/2423	involving artificial intelligence algorithms, e.g. expert systems, rule based systems, genetic algorithms
H04L 25/0254	.... {using neural network algorithms}
H04L 25/03165	.... {using neural networks}
H04L 41/16	. {Network management using artificial intelligence}
H04L 45/08	. {Learning-based routing, e.g. neural networks}
H04L 45/36	Backward learning
H04L 2012/5686	.... {Use of neural networks}
H04L 2025/03464	.... {Neural networks}
H04L 2025/03554	.... {between neural networks and tapped delay lines}
H04N 21/4662	.... {characterized by learning algorithms}
H04N 21/4663	..... {involving probabilistic networks, e.g. Bayesian networks}
H04N 21/4665	..... {involving classification methods, e.g. Decision trees}
H04N 21/4666	..... {using neural networks, e.g. processing the feedback provided by the user}
H04Q 2213/054	.Expert systems, e.g. neural networks
H04Q2213/13054	Expert system
H04Q 2213/13343	. Neural networks
H04Q 2213/343	.Neural network
H04R 25/507	... {implemented by neural network or fuzzy logic}
Y10S 128/924	Computer assisted medical diagnostics---using artificial intelligence
Y10S 128/925	Computer assisted medical diagnostics---Neural network
Y10S 706	Data processing: artificial intelligence

Patent data

We applied the search approach to patent data available in LexisNexis PatentSight. For further details and information about access, see https://www.patentsight.com/en/. It is feasible to apply the AI search method to other available patent data sources, including Patstat (Worldwide Patent Statistical Database – European Patent Office, https://www.epo.org/searching-for-patents/business/patstat.html), Derwent World Patents Index (Clarivate, https://clarivate.com/derwent/solutions/derwent-world-patent-index-dwpi/), and databases of

national patent offices (e.g., United States Patent and Trademark Office, https://www.uspto.gov/patents/search).

Reference

1. Liu N, Shapira P, Yue X. Tracking developments in artificial intelligence research: Constructing and applying a new search strategy. Scientometrics. 2021, 126, 3153-3192.
